# Effect of Nitrogen Ion Implantation on the Cavitation Erosion Resistance and Cobalt-Based Solid Solution Phase Transformations of HIPed Stellite 6

**DOI:** 10.3390/ma14092324

**Published:** 2021-04-29

**Authors:** Mirosław Szala, Dariusz Chocyk, Anna Skic, Mariusz Kamiński, Wojciech Macek, Marcin Turek

**Affiliations:** 1 Department of Materials Engineering, Faculty of Mechanical Engineering, Lublin University of Technology, Nadbystrzycka 36D, 20-618 Lublin, Poland; 2 Department of Applied Physics, Faculty of Mechanical Engineering, Lublin University of Technology, Nadbystrzycka 36D, 20-618 Lublin, Poland; d.chocyk@pollub.pl; 3 Department of Mechanical Engineering and Automatic Control, University of Life Sciences, Głęboka 28, 20-612 Lublin, Poland; anna.skic@up.lublin.pl; 4 Department of Automotive Vehicles, Faculty of Mechanical Engineering, Lublin University of Technology, Nadbystrzycka 36D, 20-618 Lublin, Poland; mariusz.kaminski@pollub.pl; 5 Opole University of Technology, Prószkowska 76, 45-758 Opole, Poland; wojciech.macek@yahoo.com; 6 Institute of Physics, Maria Curie-Sklodowska University in Lublin, pl. M. Curie-Sklodowskiej 1, 20-031 Lublin, Poland; mturek@kft.umcs.lublin.pl

**Keywords:** cavitation erosion, ion implantation, wear, failure analysis, cobalt alloy, stellite 6, damage mechanism, phase transformation.

## Abstract

From the wide range of engineering materials traditional Stellite 6 (cobalt alloy) exhibits excellent resistance to cavitation erosion (CE). Nonetheless, the influence of ion implantation of cobalt alloys on the CE behaviour has not been completely clarified by the literature. Thus, this work investigates the effect of nitrogen ion implantation (NII) of HIPed Stellite 6 on the improvement of resistance to CE. Finally, the cobalt-rich matrix phase transformations due to both NII and cavitation load were studied. The CE resistance of stellites ion-implanted by 120 keV N^+^ ions two fluences: 5 × 10^16^ cm^−2^ and 1 × 10^17^ cm^−2^ were comparatively analysed with the unimplanted stellite and AISI 304 stainless steel. CE tests were conducted according to ASTM G32 with stationary specimen method. Erosion rate curves and mean depth of erosion confirm that the nitrogen-implanted HIPed Stellite 6 two times exceeds the resistance to CE than unimplanted stellite, and has almost ten times higher CE reference than stainless steel. The X-ray diffraction (XRD) confirms that NII of HIPed Stellite 6 favours transformation of the ε(hcp) to γ(fcc) structure. Unimplanted stellite ε-rich matrix is less prone to plastic deformation than γ and consequently, increase of γ phase effectively holds carbides in cobalt matrix and prevents Cr_7_C_3_ debonding. This phenomenon elongates three times the CE incubation stage, slows erosion rate and mitigates the material loss. Metastable γ structure formed by ion implantation consumes the cavitation load for work-hardening and γ → ε martensitic transformation. In further CE stages, phases transform as for unimplanted alloy namely, the cavitation-inducted recovery process, removal of strain, dislocations resulting in increase of γ phase. The CE mechanism was investigated using a surface profilometer, atomic force microscopy, SEM-EDS and XRD. HIPed Stellite 6 wear behaviour relies on the plastic deformation of cobalt matrix, starting at Cr_7_C_3_/matrix interfaces. Once the Cr_7_C_3_ particles lose from the matrix restrain, they debond from matrix and are removed from the material. Carbides detachment creates cavitation pits which initiate cracks propagation through cobalt matrix, that leads to loss of matrix phase and as a result the CE proceeds with a detachment of massive chunk of materials.

## 1. Introduction

Cobalt-based alloys with additions of Cr, C, W, and/or Mo, named as Stellites, present superior performance in various environments such as corrosive, high temperature, wear and erosion conditions. They can well behave in advanced engineering applications such as the primary circuit in nuclear-pressurised water reactors [1], engine components [2], oil and gas applications [3], elevated temperature metal forming tools [4]. Stellites components are fabricated with various techniques, more often by overlay welding [5,6], casting [7,8] or using surface techniques likewise HVOF [9], cold spray [10], laser cladding [11], laser remelting [12] or conventional machining [13]. The functional properties of stellites depend on the fabrication process. One of the most widespread cobalt based alloy is Stellite 6 and component made of this alloy are manufactured not only by casting and welding, thermal deposition methods but also by powder metallurgy, namely hot isostatically pressing (HIP) [3,14,15,16]. Cobalt based materials are among the most cavitation erosion-resistant materials commercially available and chemical composition of Stellite 6 alloy exhibits excellent cavitation erosion (CE) resistance. Therefore, Stellite 6 superior CE behaviour is documented by many scientific papers, for the stellites produced as alloy weld overlays [17], plasma transferred arc (PTA) overlay welding [18], laser cladding [11], shielded metal arc welding (SMAW) [19] and also HVOF deposited Stellite 6 coatings [20] and cobalt-based WC-Co cermet’s deposits [21,22,23]. Unfortunately, as far as the authors’ knowledge no paper described cavitation properties of the powder metallurgy satellites and analysed the cavitation wear behaviour of HIPed Stellite 6.

Besides, there is a systematic demand to improve the operation-time of stellites by employing various post-treatment techniques presented in the literature. Thus, stellite laser remelting [24], alloying [25] or ion implantation [26] are employed to modify the properties of Co-based alloy surface layer. Even so, these processes are mostly applied for increasing stellites tribological, corrosion or erosion resistance, it should be emphasised that limited papers focus on the effect of stellites treatment on their CE resistance. Especially, ion implantation is one of the most promising anti-wear processes, which has a positive effect in a range of engineering applications such as corrosive [27] and sliding wear [26,28] performance of different metallic surfaces. Moreover, the investigation into the anti-cavitation application of different ion types and fluences into the metallic materials were discussed in the literature. It is confirmed that the ion implantation of stainless steel grade 13Cr4Ni dosed with nitrogen [29], mild carbon steel with nitrogen and titanium [30], titanium-implanted cobalt-based carbide [31] results in increasing CE resistance. Even though the literature survey confirms the positive effect of nitrogen treatment on CE behaviour of different metallic materials, the influence of the nitrogen implantation of HIPed Stellite 6 on the resistance to CE has not yet been clarified.

The Stellite alloys microstructure consists of Co-based solid solution and second phases. Cobalt matrix is usually formed by the different fraction of hexagonal close-packed (hcp) to face-centred cubic (fcc) phases, and these structures can transform under the action of temperature, strain, cobalt alloy processing, treatment, strain-affected operation conditions etc., [12,32,33]. Although the cobalt structure undergoes a phase transition from the high temperature fcc structure, stable above approx. 700 K, to the low temperature hcp phase being thermodynamically stable at room temperature, both structures are usually present. Moreover, cobalt fcc → hcp transformation is currently designated as martensitic due to its diffusionless character, its considerable thermal hysteresis and the typical nucleation and growth processes [34]. On the other hand, reverse transformation kinetics, namely the hcp → fcc phase transformation mechanism in cobalt, is still not fully explained by the literature [32,35,36]. Similarly, along with the literature survey, neither the cobalt-based solid solution phase transformation due to nitrogen ion implantation (NII) nor the effect of the CE on phase transformations of NII cobalt-matrix have been investigated. Moreover, according to the authors’ knowledge, no attention has been paid by the scientific literature to the NII effect on the CE behaviour of HIPed Stellite 6.

This work aims to investigate the nitrogen ion implantation (NII) effect on the CE resistance of HIPed Stellite 6. Besides, the effect of nitrogen dose was taken into account during the analysis of CE mechanism of HIPed Stellite 6. Finally, the cobalt-rich matrix phase transformations due to both NII and cavitation load were studied.

## 2. Materials and Methods

### 2.1. Samples Preparation, Ion-Implantation and Characterisation

The samples were machined from a round bar made of HIP-consolidated (Hot Isostatically Pressed) cobalt alloy grade Stellite 6 with diameter of 25 mm and 10 mm height. Then the flat test surface was mirror-polished to obtain the S_a_ < 0.06 µm and S_z_ < 0.8 µm, and subsequently treated with NII. Implantation was performed using ion implanter UNIMAS (Institute of Physics of Maria Curie-Skłodowska University in Lublin, Poland) equipped with arc discharge plasma ion described in [37], in configuration without any internal evaporator. Irradiations were done with 120 keV N^+^ ions with fluences 5 × 10^16^ N^+^/cm^−2^ (marked as K1 sample) and 1 × 10^17^ N^+^/cm^−2^ (marked as K2 sample) and unimplanted sample is named as K0. The distribution of implanted nitrogen ions and radiation damage (vacancies) caused by the implantation over the sample depth were performed using target (Stellite 6) chemical composition and employing the commonly used SRIM software package (version 2013, available as freeware at [38]). This binary collision approximation (BCA) method-based computer code is described in detail e.g., in [39,40]. The code enables also calculations of implantation effects on target (Frenkel pairs) using the modified Kinchin-Pease model [41]. The chemical composition of the tested alloy, given in Table 1, and measured Vickers hardness 507 ± 22 HV0.2 corresponds to literature data of Stellite 6 alloys [3,42,43].

To investigate the microstructure of implanted and unimplanted samples the scanning electron microscopy (SEM-EDS) and X-ray diffraction (XRD) methods were employed. The XRD measurements were made using the high-resolution X-ray diffractometer (Empyrean Panalytical, Almelo, The Netherlands) operated with generator voltage of 40 kV and a current of 30 mA. CuKα (λ_Cu_ = 1.5418 Å) radiation was used and analysis were performed in the θ–2θ geometry over a range from 30° to 100° with a step size of 0.01° and counting time 6 s per data point. The radiation was detected with a proportional detector. The source divergence and detector slit were 1/2, and Soller slits were applied. The crystalline phase in the samples was identified using High Score Plus software package (Version 3.0e, 2012, Panalytical BV, Almelo, The Netherlands). Finally, the crystallite sizes were calculated using the Scherrer equation given elsewhere [44]. Finally, the diffraction results were comparatively analysed.

### 2.2. Cavitation Testing and CE Damage Evaluation

CE tests were prepared using a vibratory test rig and test conditions were described in previous papers [45,46]. Cavitation was generated by a magnetostrictive-driven apparatus, resonating at 20 kHz with a peak-to-peak displacement amplitude of 50 µm. The apparatus conformed to the ASTM G-32 [47] standard recommendations, and measurements were performed by the stationary specimen method. The standoff between the sonotrode-tip and the specimen surface was set equal to 1^−0.05^ mm. The total test time lasts for 30 h. During the test, at stated time intervals, the samples were examined by precise analytical balance weighing with accuracy of 0.01 mg. The mean depth of erosion, cumulative erosion rate and incubation period were estimated. Erosion stages were read from the plotted cumulative cavitation curves. The CE results of HIPed Stellite 6 samples were compared with a popular stainless steel AISI 304, used as a reference sample.

To identify the CE wear mechanism, samples were characterised at stated test intervals using XRD, SEM-EDS, atomic force microscope (AFM) and surface profilometer. Samples surface were investigated using AFM (AFM NTEGRA Prima, NT-MDT BV, Apeldoorn, The Netherlands) on semi-contact mode using silicon cantilever NSG30 with average resonant frequency of 300 kHz. The AFM observations were conducted at stated test time intervals: 0 h, 1 h and 6 h and the CE behaviour was comparatively studied using the AFM height images (topography), deflection images (an error signal, that is the output signal from the piezoresistive bending sensor) and roughness parameters. Moreover, after 30 h of testing damaged areas were analysed using stick profilometer Form Talysurf Series 50 mm Intra (Taylor Hobson Ltd., Leicester, UK). The surface roughness parameters: arithmetical mean height (S_a_) and maximum height (S_z_) [48,49] were determined according to the ISO 25178 standard [50]. To state the effect of the cavitation load on the phase transformations, the X-ray diffraction (XRD) measurements were conducted before and after the total time of CE testing (30 h). Finally, conducted analyses allow characterizing the cavitation erosion mechanism of HiPed Stellite 6.

## 3. Results

### 3.1. Microstructure of the HIPed Stellite 6

**Figure 1:**
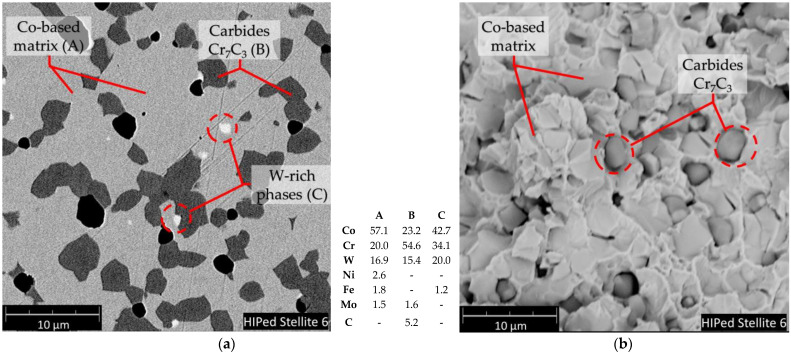
Microstructure of the HIPed Stellite 6 alloy: (**a**) polished surface and chemical composition of A, B and C spots; (**b**) structure of fractured sample, SEM-EDS.

Literature survey reports that HIPed stellites microstructure consists of cobalt-based alloy metallic matrix and second phases, mainly hard chromium carbides [51,52,53]. This is also confirmed by our study involving, the chemical composition analysis, metallographic and X-ray diffraction investigations which confirmed the HIPed Stellite 6. As-received K0 sample microstructure contains cobalt-based solid solution matrix consisting of Co-Cr solid solution alloyed with tungsten, nickel, iron and molybdenum (Figure 1, spot A) as well as other phases mostly carbides (Figure 1, spots B and C).

**Figure 2:**
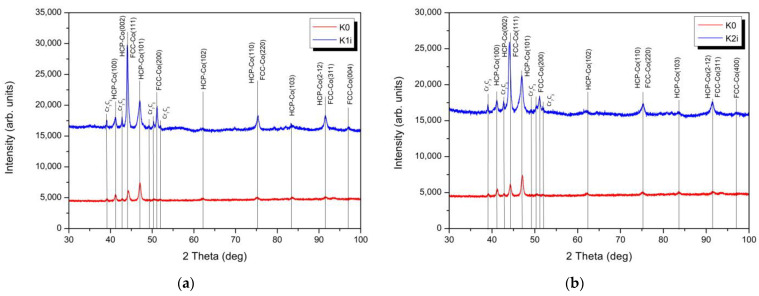
The X-ray diffraction (XRD) patterns of a HIPed Stellite 6 before (red line) and after implantation (blue line) with two different doses: (**a**) 5 × 10^16^ N^+^/cm^−2^ (sample K1i) (**b**) 1 × 10^17^ N^+^/cm^−2^ (sample K2i).

Moreover, XRD patterns presented in Figure 2 show that the HIPed stellite 6 matrix exhibits two crystal structures, γ (fcc—face-centred cubic) and ε (hcp—hexagonal close-packed) phases. The ratios of fcc to hcp phases can be influenced by NII-induced phase transformations. Figure 2 shows the change in XRD profile for samples implanted with nitrogen ions of two different doses (samples K1 and K2) compared to unimplanted sample (K0). 

In both cases, we can see the same and significant changes. For unimplanted sample, in XRD profile we can distinguish high intensity peaks at 2θ values of 41.18°, 44.24° and 47.03° corresponding to (100), (002) and (101) planes of hcp phase and a very weak peak at 2θ values of 51.12° from (200) planes of fcc phase. The presence of the fcc phase in the unimplanted sample is also indicated by an increase in intensity peaks at 2θ values of about 44° due to the overlap of peaks corresponding to the hcp (002) and fcc (111) planes. In case of the implanted samples (K1 and K2), XRD profiles revealed a significant increase in peak’s intensity at 2θ values of about 44°, 51°, 75° and 91.5°. The increase in intensity of peak at 2θ values of about 51° is directly related to the growth of fcc phase (some hcp reverts to metastable fcc). Furthermore, the increase in the intensity of the remaining peaks is related to the growth of the areas with fcc structure in the solid solution and the overlapping of the peaks from the planes: hcp (002) and fcc (111); hcp (110) and fcc (220), and hcp (212) and fcc (311), respectively (see Figure 2). In relation to a lower dose of ions (K1), increase in the intensity of these peaks is greater, consequently, the hcp to fcc ratios of K1 exceeds those reported for K2. The accumulation of structural defects produced during implantation results in the absorption of energy by the material and facilitates the hcp to fcc transformation [54]. This can be clarified by recovery and removal of strain, dislocations and phase transformation hcp → fcc under implantation process (Figure 2). Regarding a lower dose of ions, the increase in the intensity of these three peaks is greater, which indicates that the hcp phase is first transformed to fcc phase (dose 5 × 10^16^ N^+^, Figure 2a) and subsequently dosed until 1 × 10^17^ N^+^, it is converted back to hcp phase due to absorption of higher energy (Figure 2b). Additionally, the ion implantation modifies the crystallite grain size, which is confirmed by broadening the XRD diffraction peaks. From the analysis of the half-widths of the peaks for peaks that do not overlap, it can be concluded that during implantation there is a refinement of the hcp grains size from 17.9 nm (K0) to 8.8 nm and 10.4 nm for a lower and higher dose of ions, respectively. At the same time, contrary to unimplanted sample, growth of grains with the fcc structure was observed for a lower dose of ions (K1), with a size of 15.1 nm (K1), in comparison to grain size 11.0 nm for a higher dose of ions (K2). Furthermore, Houdková et al. [12], who studied different surface treatments of Stellite 6, confirmed that variation of hcp to fcc-based phase’s ratio depends on the type of deposition and treatment processes. Thus, the powder metallurgy manufactured HIPed Stellite 6 phase composition may be expected to differ from those reported for welded or thermally deposited stellites.

**Figure 3:**
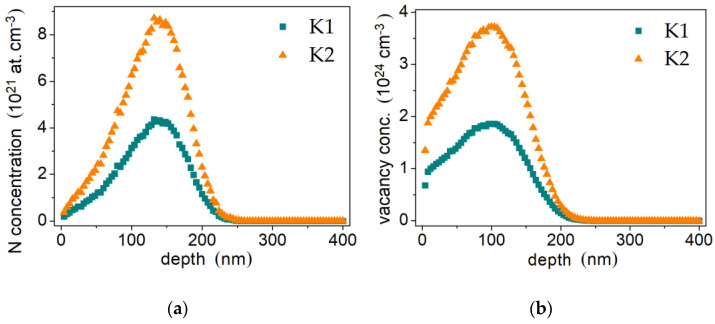
Theoretical distribution of implanted by 120 keV nitrogen ions with different nitrogen doses: (**a**) K1: 5 × 10^16^ N^+^/cm^−2^ and K2: 1 × 10^17^ N^+^/cm^−2^ (**b**); implantation-generated vacancies (radiation defects).

The XRD confirms that dominant microstructure secondary phase is a chromium carbide Cr_7_C_3_ (indicated by the light grey areas in Figure 1, spot B), besides the structure contains a small amount of tungsten-enriched phases (white areas, Figure 1, spot C). The ion implantation affects the changes of cobalt-based solid solution crystallite structure. Exemplary in the case of aluminium alloy literature reports [55] that high-dose ion implantation of nitrogen leads to the formation of nitride phases which is a result of chemical bond rearrangement. However, no nitride phases were confirmed by the XRD investigation of Stellite 6 (Figure 2). The NII results mainly in a change of hcp to fcc structure. Figure 3 presents the distribution of implanted nitrogen ions and radiation damage (vacancies) caused by the implantation over the sample depth. It is well seen that the higher nitrogen dose results in a two-fold higher concentration of nitrogen ions, and the maximum concentration is observed at approx. 145 nm depth from the top surface (Figure 3a) while vacancies defects density is highest at approx. 100 nm depth (Figure 3b). Hcp to fcc phase’s ratio depends on implantation fluences. Also, the radiation defects have the maximum concentration at 100 nm depth (Figure 3b). On the other hand, it is reported by the literature [56,57,58] that the long-range effect of ion implantation could exceed the range of nitrogen ions-implanted zone. Budzyński et al. [26] claimed the nitrogen ion-affected zone of Stellite 6 is four times thicker than the depth of the defects, reaching about 1.0 µm thickness of the layer. This long-range effect supports the structural modification in the subsurface layer at NII and could be manifested in increase of the dislocation density [57]. To conclude, the nitrogen interaction facilitates the hcp transformation into fcc in the ion-affected subsurface zone of HIPed Stellite 6.

### 3.2. Cavitation Erosion (CE) Resistance

**Figure 4:**
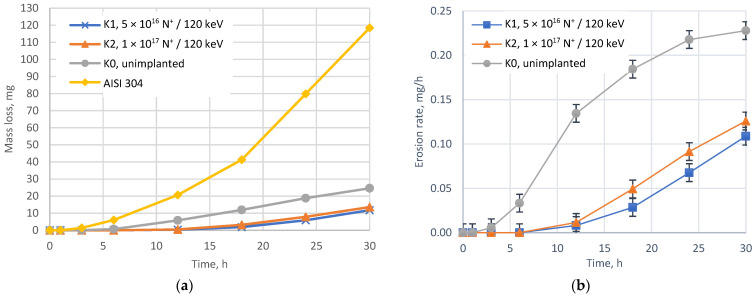
Cavitation erosion (CE) curves of nitrogen-implanted and unimplanted HIPed Stellite 6 and reference stainless steel (AISI 304) cumulative mass loss (**a**); (**b**) cumulative erosion rate curves of HIPed Stellite 6.

The cavitation erosion curves of unimplanted and nitrogen-implanted HIPed Stellite 6 and reference stainless steel are presented in Figure 4 while the summary of CE indicators is displayed in Table 2.

**Figure 5:**
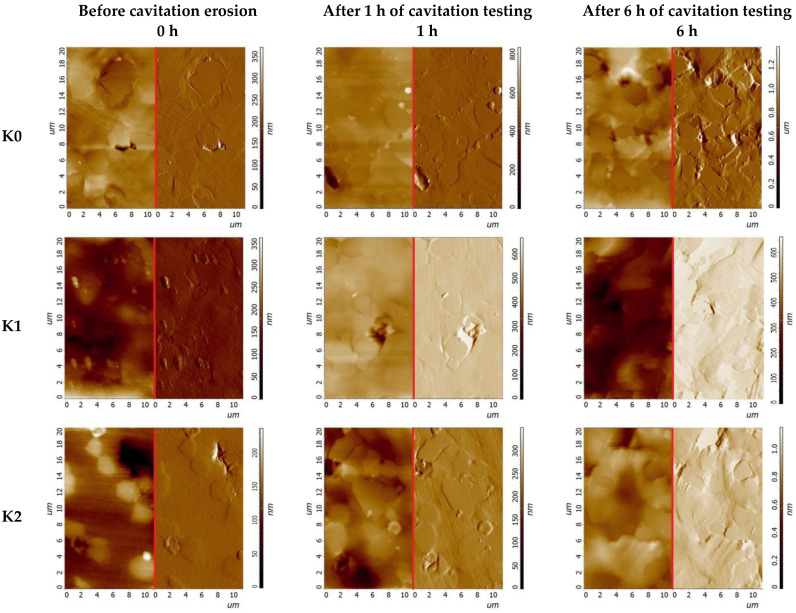
Surface morphology development in the initial stage of CE of HIPed Stellite 6. Samples K0, K1 and K2 studied at 0 h, 1 h and 6 h of cavitation testing. Surface morphology height images (**left**) and corresponding deflection images (**right**), AFM.

**Figure 6:**
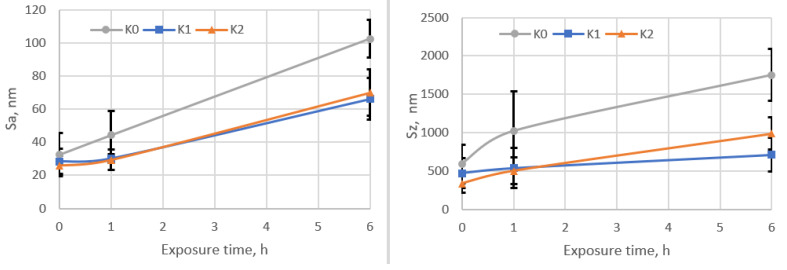
Areal roughness S_a_ and S_z_ parameters measured in initial stage of cavitation erosion.

**Figure 7:**
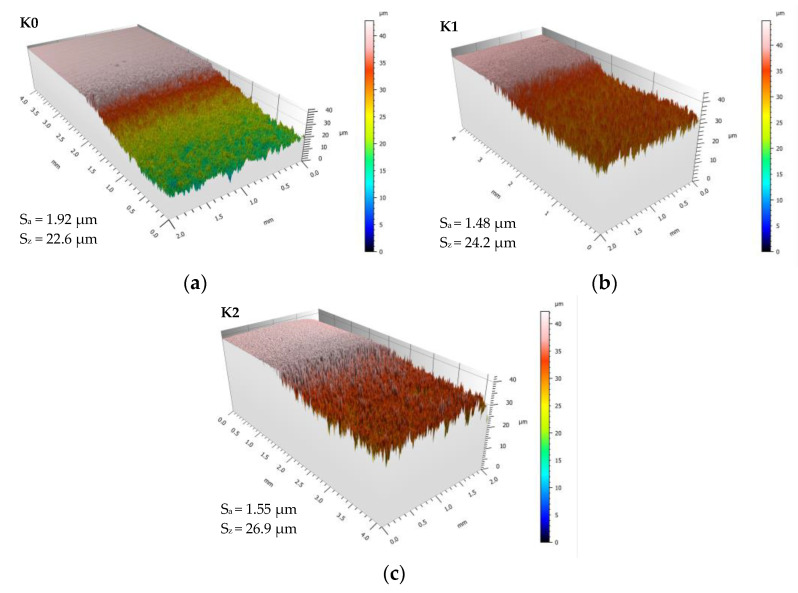
Surface morphology development: undamaged area and area of samples after 30 h of cavitation testing: (**a**) unimplanted: K0–K0c; (**b**) implanted with a fluence of 5 × 10^16^ N^+^/cm^2^: K1–K1c; (**c**) implanted with a fluence of 1 × 10^17^ N^+^/cm^2^: K2–K2c.

Figure 5, Figure 6 and Figure 7 compares surface morphologies at stated CE testing time intervals. In addition to this, Figure 6 presents the quantitative areal roughness S_a_ and S_z_ parameters vs. exposure time.

The NII plays an important role in controlling the CE behaviour of HIPed Stellite 6. Analysis of quantitative results confirms that the implanted HIPed Stellite 6 has almost ten times higher resistance to CE than the stainless steel grade AISI 304. It should be noted that unimplanted stellite CE resistance is only five-times higher than the reference AISI 304 sample. Additionally, the CE results analysis proves that the NII three times elongates the incubation period of erosion and successively mitigates the erosion rate, and decreases mass loss and mean erosion depth, see Table 2 and Figure 4.

Furthermore, cavitation results follow the XRD results which confirm that K1 sample presents higher fcc content and higher CE resistance than K2 sample. It should be noted that the transformation of hcp to fcc structure, caused by the nitrogen ion-implantation (Figure 2), is essential to mitigate the erosion damage, especially in the incubation period of CE. This is supported by the analysis of surface morphology development studied using AFM, see Figure 5 and Figure 6. The comparative analysis of the height images with deflection images clarifies the HIPed Stellite 6 surface morphology development and explains the cobalt matrix time-dependent CE behaviour due to cavitation loads. The unimplanted K0 microstructure is dominated by the hcp phase which has higher stacking fault energy [59] than fcc, thus it decreases the ability to work-hardening and affects matrix detachment at the carbide/ solid solution interfaces. Consequently, due to cavitation loads, the surface of K0 exhibits higher roughness than implanted samples. The nitrogen-treated surfaces of samples K1 and K2 (rich in fcc) are less prone to cavitation and as a result, have lower roughness. It is known that the hcp structure has a lower ability to work hardening and is usually tougher than the fcc crystalline structure. The fcc structure is metastable at room temperature and can be transformed into hcp under strain-inducted transformation [54,60] due to cavitation. The phase transformation consumes the energy generated by cavitation for the phase fcc → hcp and effectively decreasing the surface damage.

Changes in the surface morphology, as well as the development of S_a_ and S_z_ roughness parameters (Figure 5, Figure 6 and Figure 7), well correlates with the CE results given in Figure 4. Comparable analysis of the roughness parameters during the initial stage of erosion done for the unimplanted sample K0, with nitrogen-implanted samples (K1 and K2), visualised that ion-treated samples display a lower rate of surface roughening, and at the end of incubation period, the K1 sample obtains the lowest roughness, Figure 6 also elsewhere in text. This agrees with the surfaces morphology visualised at stated time-exposure, for 1 and 6 h in Figure 5. Besides, the 30 h roughness of unimplanted sample K0 has higher values of S_a_ = 1.92 µm and lower S_z_ = 22.6 µm than estimated for nitrogen-implanted K1 (S_a_ = 1.48 µm, S_z_ = 24.2 µm) and K2 (S_a_ = 1.55 µm, S_z_ = 26.9 µm). It seems that the arithmetical mean height (S_a_) parameter well compares to the sample’s damage rate order while the maximum height (S_z_) provides information about the damage mechanism because it refers to the sum of the roughness maximum peak height and maximum valley depth. Finally, at 30 h of cavitation testing K0 presents accelerated stage of erosion and high rate of material removal than NII samples K1 and K2 which are in the earlier stage of CE affected rather by pitting and craters formation (increasing S_z_) than on uniform mode removal, explained by less advanced material removal and lower mean roughness (S_a_).

**Figure 8:**
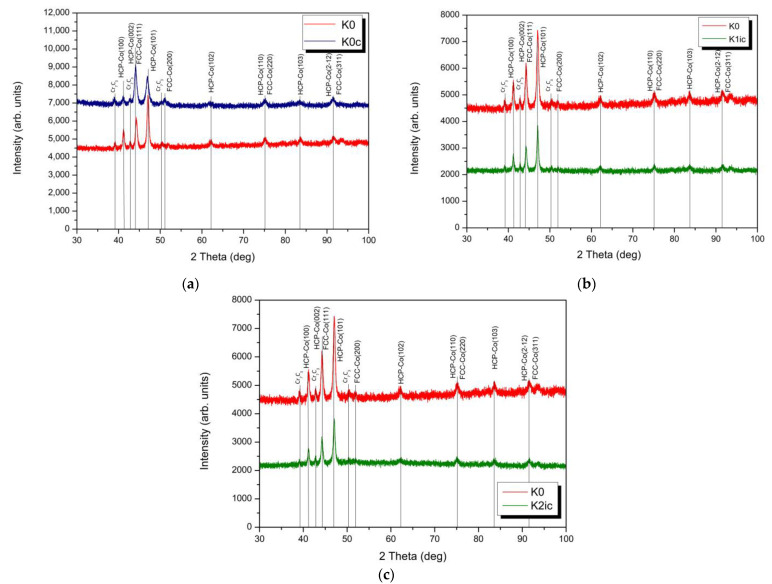
X-ray diffraction patterns obtained for HIPed Stellite 6 samples before and after 30 h exposure to cavitation of (**a**) unimplanted: (**b**) nitrogen implanted with dose of 5 × 10^16^ N^+^/cm^2^ (K1ic) and (**c**) nitrogen implanted with dose of 1 × 10^17^ N^+^/cm^2^ (K2ic).

Comparison of the cavitation effect on the X-ray profiles for unimplanted and implanted samples after 30 h of cavitation testing (labels ended with “c”; K0c, K1ic, K2ic) are presented in Figure 8.

The XRD analysis suggests that the eroded surfaces of K1ic and K2ic (investigated after 30 h) display the presence of both hcp and fcc structures, comparable to unimplanted K0 (before cavitation erosion testing). Although the proportion of the hcp to fcc for K1c and K2c differs from those identified before cavitation testing, see Figure 2. In case of the K0c sample, it seems that the tougher and dominated by hcp surface layer of K0 poorly prevents the underneath material from degradation. In addition to this, the CE of unimplanted K0c manifests in hcp → fcc phase transformation, see Figure 8a. Generally, the influence of cavitation on solid surfaces is described as a combination of mechanical and temperature fields precisely, by the mechanical action of the collapsing cavitation bubbles and microjets [61,62] which have a fatigue nature [63,64] as well as by a high-temperature field generated during cavitation phenomenon [65,66]. It is claimed by the literature that elevated temperature affects the recovery process in the crystalline structure of CoCr alloy [67]. While increasing the temperature of pure cobalt, at 417 °C the hcp structure undergoes phase transformation to the high temperature fcc form [68].

The cavitation-load of implanted samples K1ic and K2ic results in metastable fcc to hcp transformation. It seems load is consumed for the strain-induced fcc → hcp martensitic transformation, see Figure 8 (cavitation-load induces phase transformation [12]). This phenomenon is well documented in the case of unimplanted stellite alloys subjected to cavitation loads [69] and mechanical action likewise sliding wear at room and elevated temperatures [59]. Besides after 30 h of the testing, in the case of K1ic and K2ic samples higher content of hcp was confirmed than for unimplanted sample K0c (indicated by lower XRD peaks intensity, see Figure 8). This can be explained by the proceeding martensitic transformation in the case of K1c and K2c samples. Moreover, the effect of nitrogen ion fluences on the development of cavitated structure has been confirmed. The K1c sample is much more prone to fcc formation than the K2c sample. This refers to the differences in nitrogen doses and higher fcc content after ion-implantation observed for K1 sample, see Figure 2. A higher nitrogen fluence reduces the cobalt-based solid solution deformability and facilitates carbides debonding, see Figure 8b,c, as a result, ends up with the increase of CE damage of the K2 sample.

**Figure 9:**
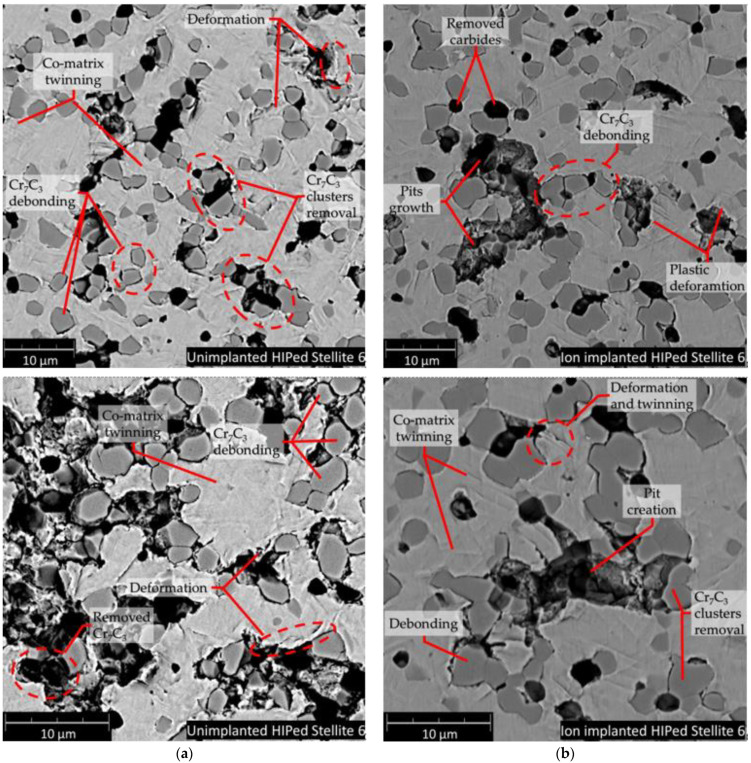
The initial stage of cavitation erosion of HIPed Stellite 6: (**a**) unimplanted sample (**b**) nitrogen implanted, SEM.

**Figure 10:**
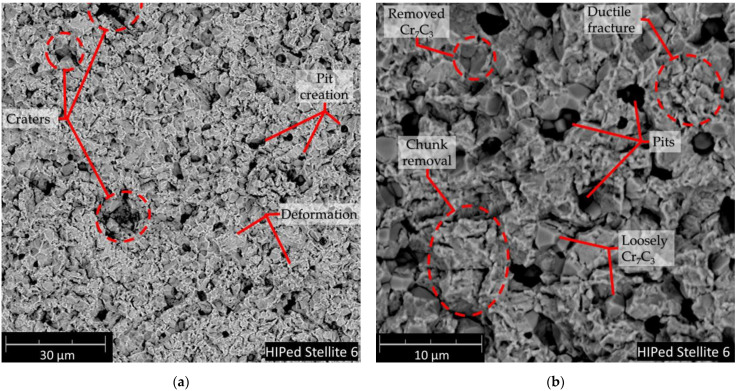
Damaged surface of HIPed Stellite 6 at 30 h of exposure: (**a**) surface overview (**b**) enlarged area, SEM.

Undoubtedly, CE affects the ion-implanted HIPed Stellite 6 behaviour mainly, by elongating the incubation period of erosion and bonding the carbides in the cobalt matrix. Thus, in the later stages of CE, the fcc structure formed due to ion implantation transform back to hcp phase (see diffractograms of K1c and K2c samples). Moreover, the martensitic transformation ability reduced (due to fcc depletion) and, severe surface roughening (see Figure 7) and debonding of Cr_7_C_3_ was initiated resulting in pit formation, see Figure 9 and Figure 10). These mechanisms favour detachment of the material end exposure of deeper located hcp structures. Thus, we believed that nitrogen-implanted samples in longer than 30 h exposure testing, due to the exposure of the deeper located unimplanted bare material, should behave as K0c sample and hcp → fcc transformation is expected (as unimplanted stellite). Summing up, all these factors contribute to the elongation of the incubation period, reduces the damage rate of implanted surfaces and overall positively donates for CER of nitrogen-dosed HIPed Stellite 6.

### 3.3. Cavitation Erosion Mechanism of HIPed Stellite 6

The SEM analysis of cavitation worn surfaces (see Figure 9 and Figure 10), surface morphology analyses, XRD phase investigations allow to state the erosion mechanism. Generally, the HIPed Stellite 6 initial microstructure can be simplified to two dominant phases, cobalt-based matrix and Cr_3_C_7_ carbides. Therefore, the CE wear mechanism of HIPed stellite 6 starts at cobalt-rich matrix surface roughening due to plastic deformation of (Figure 5 and Figure 6). The cobalt-based solid solution exhibits twining but much severe deformation is observed in hcp-rich phase K0 sample than for nitrogen-doped K1 and K2 surfaces (Figure 9 and Figure 10).

Twinning is a common deformation mechanism in hcp metal and alloys subjected to plastic deformation [70]. XRD investigation confirmed that nitrogen implantation of Stellite 6 provides increase of fcc crystallites, and the presence of this phase elongates the initial period of erosion, Table 2. The AFM areal roughness measurements confirmed (Figure 5 and Figure 6) that the NII results in less harsh roughness development. Furthermore, fcc crystallite is prone to plastic deformation and has higher deformability than hcp and consequently prevents debonding at cobalt matrix and Cr_3_C_7_ interfaces (Figure 9).

This mechanism elongates the CE incubation stage of N-implanted Stellite 6. Further, deformed metallic matrix contributes to ceramic phase losses along with the ceramic clusters which first undergoes spallation. At the interfaces of hard carbide particles and cobalt-based solid solution, the voids arose, and plastic deformation of the cobalt-matrix is visible in Figure 9. This weakens the carbide restrain and material detachment starts at the ceramic particles removal. Pits and dimples after the carbide removal are created Pit edges, exposed to CE lose their support, are plastically deformed and detach finally. The debonding and cracking in Cr_7_C_3_ results in spallation of the hard carbides and introduces pits which are the centre of material cracking, deformation and accelerated degradation. It should be pointed out that this damage mechanism proceeds at a lower rate for nitrogen-implanted stellites, see Figure 9. We have noticed that the initial stage erosion behaviour of the HIPed Stellite 6 is comparable to those observed for MMC (metal matrix composites) reported in our previous study [71] for Al/Al_2_O_3_ and Cu/Al_2_O_3_ cold sprayed MMC’s, mainly due to semi-metallic/ceramic microstructure of HIPed Stellite 6 which relates to the manufacturing process (powder metallurgy). During later periods of cavitation ion-affected zone is also removed (Figure 10). Moreover, massive material chunks are losing restrain. Erosion progresses in the deeper located unchanged/fresh structure of the material is exposed to deterioration. The cobalt matrix erosion proceeds in plastic deformation mode. The XRD analyses confirm that implanted K1 and K2 samples undergo martensitic transformation. As it was mentioned earlier in the text, it is expected that in further longer exposure time, implanted samples K1 and K2 undergo martensitic transformation comparable to unimplanted K0 sample.

Summing up, the proposed cavitation erosion model differs from those presented for welded stellites [17] or HVOF-deposited WC-CoCr cermet’s [72]. In addition, this paper preliminary signalises the effect of NII on reinforcement of carbides bonding to cobalt matrix by a decrease of hcp to fcc ratio. This initial strengthening of metallic matrix reduces erosion rate due to blocking of Cr_3_C_7_ particles detachment and has been associated with elongation of incubation stage, consequently improving CE resistance of HIPed Stellite 6.

## 4. Conclusions

This work discusses the nitrogen ion implantation effect on the cavitation erosion (CE) resistance and phase transformations of HIPed Stellite 6 cobalt alloy. Also, the effect of nitrogen dose was taken into account during the analysis of CE mechanism of HIPed stellite 6. Finally, the influence of cavitation on phase transformation of ion-implanted cobalt-based solid solution was preliminarily discussed. The following conclusions are drawn:

1. Ion-implanted of HIPed Stellite 6 by 120 keV with fluences 5 × 10^16^ N^+^/cm^−2^ and 1 × 10^17^ N^+^/cm^−2^ increases the CE resistance, finally by two times decreases the material loss, three times elongating the incubation period of erosion than for unimplanted stellite and has almost ten times higher CE resistance than a popular reference sample made of AISI 304 stainless steel.
2. The dose-effect on CE has been confirmed. Unimplanted HIPed Stellite 6 presents severe erosion due to hcp cobalt matrix decreasing the alloy deformation ability and bonding of Cr_7_C_3_. Nitrogen ion implantation results on ε(hcp) → γ(fcc) transformation and fcc structure facilitates CE resistance. Stellite dosed with lower fluences of 5 × 10^16^ N^+^/cm^−2^ has a lower hcp to fcc ratio and consequently presents increasing resistance to CE.
3. Nitrogen ion implantation of HIPed Stellite 6 strengthens the cobalt-solid solution by fcc structure formation which effectively bonds Cr_7_C_3_ carbides in matrix, mitigates the matrix ductile fracture, finally increasing the CE incubation time and decreasing erosion rate.
4. Nitrogen ion implantation initiates in hcp phase recovery process, removal of strain and dislocations and facilitates fcc phase formation. Under the cavitation-loads, nitrogen-implanted HIPed Stellite 6 fcc phases well-consume the cavitation load for work-hardening and Co-based matrix martensitic fcc → hcp transformation. All those transformations beneficially contribute to decreasing the cavitation rate of nitrogen-implanted stellite.
5. The CE wear mechanism of HIPed Stellite 6 relies on the plastic deformation of the cobalt matrix starting at Cr_7_C_3_ and Co-matrix interfaces. Once the chromium carbides lose their restrain in the matrix, debond and are removed. Carbides detachment creates cavitation pits which initiate cracks propagation through cobalt matrix which ends with a detachment of massive chunk of materials.
6. Undoubtedly, nitrogen ion-implantation of HIPed Stellite 6 has a beneficial effect on their CE resistance and in the future, the nitrogen ion dose and implantation energy should be optimised to form the ideal ratio of hcp to fcc in cobalt-based solid solution.

## Figures and Tables

**Table 1:** The chemical composition of HIPed Stellite 6.

Chemical Composition of HIPed Stellite 6 *, wt%								
Co	Cr	W	C	Fe	Ni	Si	Mn	Mo
bal.	28.40	6.15	1.34	2.00	2.18	0.60	0.45	1.45

* Measured using XRF.

**Table 2:** Summary of the cavitation erosion results of stellites and reference stainless steel.

Sample	Incubation Time, h	Cumulative Erosion Rate, mg/h	Mean Erosion Depth_max_, µm
AISI 304	1.3	1.10	78.11
K0	2.0	0.23	15.26
K1	6.0	0.11	7.29
K2	6.0	0.13	8.43

## Data Availability

Data is contained within the article.
